# Development of hybrid green nanocomposite polymeric beads doped with nano sulfated zirconia for effective removal of Cefotaxime antibiotic from aqueous solution

**DOI:** 10.1038/s41598-022-16473-z

**Published:** 2022-07-26

**Authors:** Marwa H. Gouda, Noha A. Elessawy, Arafat Toghan

**Affiliations:** 1 Polymer Materials Research Department, Advanced Technology and New Materials Research Institute (ATNMRI), City of Scientific Research and Technological Applications City (SRTA-City), Alexandria, 21934 Egypt; 2 Computer Based Engineering Applications Department, Informatics Research Institute IRI, City of Scientific Research & Technological Applications (SRTA-City), Alexandria, 21934 Egypt; 3 Chemistry Department, Faculty of Science, South Valley University, Qena, 83523 Egypt; 4 Chemistry Department, College of Science, Imam Mohammad Ibn Saud Islamic University (IMSIU), Riyadh, 11623 Saudi Arabia

**Keywords:** Environmental sciences, Engineering

## Abstract

Adsorption efficiency of *Cefotaxime* by novel nanocomposites beads composed of iota carrageenan (IC), sulfonated poly vinyl alcohol (SPVA) and nano sulfated zirconia **(**SZrO_2_) was evaluated in this study. SZrO_2_ was synthesized from solvent-free and easy calcination technique then embedded with 1–2.5 wt.% into the polymeric matrix. A batch adsorption experiment was carried out to investigate the effects of dosage, pH, beginning concentration, and time on *Cefotaxime* antibiotic adsorption. The ideal conditions to achieve complete removal are 88.97 mg L^−1^ initial cefotaxime concentration at time 3.58 h with 11.68 mg of beads composite with 2.5 wt.% of SZrO_2_. The pseudo second order kinetics model better illustrated the adsorption of cefotaxime on nanocomposite beads, and the maximum adsorption capacity are 659 mg g^−1^ for the composite with 2.5 wt.% of SZrO_2_. The mechanism of adsorption process depend mainly on the interactions between the different functional groups of SPVA, IC and SZrO_2_. The nanocomposites beads also exhibit excellent reproducibility after ten adsorption cycles. This type of nanocomposites beads can be easily separated from water without leaving any residue, verifying this novel nanocomposite beads has strong potential in water treatment for the antibiotic contaminant removal.

## Introduction

Water treatment processes confront significant challenges in terms of optimizing technology to avoid human health concerns and to ensure environmental sustainability, as population grows, water sources become scarcer and water quality deteriorates due to land use and climate change^1^. These water-related issues are better understood and handled as a result of enhanced detection and awareness of the toxicological, environmental, and biological effects of an ever-growing list of substances dubbed Emerging Pollutants (EPs). EPs are a group of man-made chemicals, such as pharmaceuticals, pesticides, flame retardants, detergents, and cosmetics, that are essential to contemporary living and whose manufacture and use have risen dramatically in the previous decades^1–3^. The uncontrolled and continuous release of these new compounds into the environment may have an impact on aquatic biota, human health^4,5^ and also the performance and costs of drinking water treatment technologies^6,7^.

Antibiotics are classified as a type of persistent pollutant that lead to fast spread of different types of resistant bacteria. *Cefotaxime* is important type of cephalosporin antibiotics for the treatment of gram negative and positive bacterial infections with a lengthy half-life owing to its aromatic-ring structure. It is usually detected in wastewater and drinking water and is very difficult to totally remove^8^. Nowadays, there are different techniques have been applied to eliminate antibiotics from aquatic system, such as oxidation^9^, adsorption and degradation^10,11^. Between these techniques, adsorption is cheap, simple, promising method for antibiotics, organic and inorganic contaminants removal from water^12–15^. The polymeric beads as adsorbent have been more favorable than the nanoparticles, film or fiber forms because of its easy fabrication, excellent dispersion, controllable size dimension and quick recovery process.

Carrageenan is a sulfated polysaccharide made up of anhydrogalactose and galactose units found in red seaweed extracts^16^. Iota carrageenan is the most often utilized, which comprise two sulfated groups per disaccharide unit. Because of its chemical functionality and natural abundance, it is widely used for water treatment, namely in the removal of pharmaceutical contaminants and pesticides^17^.

Poly vinyl alcohol (PVA) is an eco-friendly, biodegradable and cost-effective polymer with outstanding chemical stability and hydrophilicity properties; therefore, it is widely used to eliminate various pollutant from water. This type of polymer contains abundant hydroxyl groups and can be easily coagulated and cross-linked, however, by mixing PVA with IC lead to create porous network rich with hydroxyl and sulfate groups, which can be effective for the adsorption capacity enhancement^18^.

The presence of metal oxide nanostructures in the surface and structure of polymeric matrix leads to the production of nanocomposite polymeric-based matrix, which has been proposed as one of the most potent tools for preventing membrane fouling in various water treatment processes^19–23^. Among metal oxide nanoparticles, ZrO_2_ has received a lot of interest in recent years due to its unique properties such as good chemical and mechanical stability with high hydrophilic properties, in addition to their availability, low cost and simple synthesis method^21^. However, sulfated zirconia as the adsorbent consider as a cation exchanger that promotes more elimination of the cationic counterpart such as wide sector of antibiotics which contain amine groups.

This work aimed to synthesis eco-friendly and effective green adsorbent nanocomposite beads prepared by using water as a solvent to process biodegradable and low-cost polymers to remove *Cefotaxime* antibiotic contaminant from water. PVA was chosen as the key polymer in the beads due to its good interaction with IC polymer to form compatible composite rich with functional groups, in addition to supporting SZrO_2_ nanoparticles. To our knowledge, this is the first time to SZrO_2_ nanoparticles have been incorporated into PVA/IC blending polymers matrix in different concentrations to create nanocomposite beads named as SPVA/IC/SZrO_2_ and use it as adsorbent for antibiotics in water treatment.

## Experimental

Iota carrageenan (type V), PVA (99% hydrolysis and medium MW), *Cefotaxime* sodium salt and 4-sulphophthalic acid (SPA) (99.9 wt.% in H_2_O) were purchased from (Sigma-Aldrich, USA) and Glutaraldehyde (GA) (50 wt.% in H_2_O) was purchased from Alfa Aesar.

### Synthesize

#### Synthesize of nano-sulfated zirconia (SZrO_2_)

Nano SZrO_2_ was prepared using a simple calcination method in the absence of any solvent between zirconium oxychloride octahydrate ZrOCl_2._8H_2_O and ammonium sulfate (NH_4_)_2_SO_4_ with 1:5 molar ratio were mixed and ground in a mortar then placement for 12 h at room temperature, after that calcined for 5 h at 600 °C. Finally, the powder was ground in ball mill at 1500 rpm for 30 min to obtain nano size.

#### *Preparation of SPVA/IC/SZrO*_*2*_* beads*

First, PVA and IC were dissolved separately in 100 mL deionized H_2_O at 90 °C for 2 h then blending with percentage PVA: IC (95:5) wt.%. After that, crosslinking the polymers blend by certain amount of GA as covalent crosslinker and SPA as ionic crosslinker and sulfonating agent for PVA, to convert to sulfonated polyvinyl alcohol (SPVA)^22^. Then the prepared nanocomposite was incorporated with different concentrations of SZrO_2_ (1wt.% and 2.5wt.%) in polymeric blend. The two solution samples were named SPVA/IC/SZrO_2_-1, SPVA/IC/SZrO_2_-2.5. The composite solution was put in syringe, dropped in saturated solution of boric acid to form beads, and left for overnight then washing.

As illustrated in Fig. S1 in supplementary information which shows the possible structure of SPVA/ IC/ SZrO_2_ beads, esterification reactions between carboxylic groups of SPA and hydroxyl groups of two polymers were used to ionically crosslink IC and PVA. Furthermore, acetal interactions between GA's aldehyde groups and the hydroxyl groups of the two polymers covalently crosslinked the two polymers. While SZrO_2_ was bonded to polymeric matrix by hydrogen bond interactions between its oxygenated groups and the -OH groups of two polymers.

#### Characterization

The characteristic groups of SZrO_2_ powder and the nanocomposite beads were monitored by Fourier transform infrared spectrophotometer (Schimadzu FTIR-8400 S-Japan), while the structures were evaluated by X-ray diffractometer (Schimadzu7000-Japan). Thermal changes of SPVA/IC/SZrO_2_ beads were traced by using thermo-gravimetric analyzer (Shimadzu TGA-50, Japan) the range of the temperature was 25–800 °C, under nitrogen atmosphere and the heating rate at 10 °C min^−1^. Morphological structure of the SPVA/IC/SZrO_2_-2.5 beads was shown by scanning electron microscope (SEM) combined with energy-dispersive analysis X-ray (EDX) (Joel Jsm 6360LA-Japan). Visualization of the nano SZrO_2_ was done by using transmission electron microscopy (TEM, JEM 2100 electron microscope).

To investigate the beads stability, pieces with diameter of 1 mm were immersed in 100 mL of deionized water at 35℃ for 24 h. The swelling coefficient S was calculated as follows:

1 $$\mathrm{S}\left(\mathrm{\%}\right)=\frac{{\mathrm{W}}_{w}-{\mathrm{W}}_{d}}{{\mathrm{W}}_{d}} \times 100$$

where *W*_*w*_ and *W*_*d*_ were the masses of the swollen and dry PVA/IC/SZrO_2_ nanocomposite bead, respectively.

To determine the point of zero charge (pHpzc) of PVA/IC/SZrO_2_ nanocomposite bead, 5 mg of beads were added up to 10 mL of solutions containing the pH values ranging from 3 to 9. The suspensions were then shaken gently for 24 h, then final pH values of every solution were recorded and the pH_PZC_ values was calculated.

#### Batch adsorption tests

Adsorption tests were carried out by using a batch equilibration technique. One gram per liter stock solutions of *Cefotaxime* antibiotic were diluted to different degrees by adding deionized water. However, 50 mL of *Cefotaxime* antibiotic solutions with different concentrations (25, 50,100 and150 mg L^-1^) at 25 ˚C were added with a specific amount of prepared nanocomposite beads, with the help of a thermostated shaker running at 150 rpm. After reaching equilibrium, the adsorbent beads were separated and the remaining antibiotic concentration in the aqueous phase was measured using a UV spectrophotometer at 235 nm.

The amounts of adsorbed antibiotic were calculated according to the following formulas:

2 $${q}_{t}=\frac{{C}_{0}-{C}_{t}}{m} V$$

3 $${q}_{e}=\frac{{C}_{o}- {C}_{e}}{m} V$$

where qt and qe (mg g^−1^) were the amounts of antibiotic adsorbed per unit weight of adsorbent at time t and equilibrium; C_0_, C_t_ and C_e_ mg L^−1^ were the antibiotic concentrations at initial time, time t and the equilibrium time, respectively; V (L) was the volume of antibiotic solution; and m (g) was the amount of adsorbent. The removal efficiency R was determined as follows:

4 $$R\left(\%\right)=\frac{{C}_{0}- {C}_{t}}{{C}_{0}}\times 100$$

To optimize the *Cefotaxime* antibiotic removal conditions, we tried to establish a relationship between factors and responses, according to a response surface methodology (RSM) model. The selected matrix for the response surface methodology followed the Box-Behnken design^24^, with 17 trials. To evaluate the adsorption process performance, three factors were used: A (time, min); B (initial antibiotic concentration, mg L^−1^); and C (beads adsorbent dose, mg), at three levels illustrated in Table S1 in supplementary information. Design-Expert, 13.0.9.0 programme from STAT-EASE, INC was used for experimental design, model construction and data analysis. After proper optimization of the adsorption, the kinetic and isotherm parameters of the process were characterized.

### Static kinetics and isotherms models of adsorption

The rate and mechanism of the adsorption process could be elucidated based on kinetic studies with different initial concentrations of *Cefotaxime* antibiotic varied from 25 to 150 mg L^−1^. The pH was fixed at 5 and dose of adsorbent was 2 mg mL^−1^, while the adsorption time was varied from 0 to 24 h. In order to elucidate the adsorption kinetics, the pseudo-first-order, pseudo second-order and intraparticle diffusion models were applied, as illustrated in Table 1. Additionally, to describe how the adsorbate interacts with adsorbents and give a thorough understanding of the nature of interaction, the adsorption isotherm models were tested to validate the antibiotic uptake behavior of PVA/IC/SZrO_2_ composite bead using Langmuir and Freundlich isotherms.

**Table 1:** Kinetics and isotherms models of adsorption process.

Model	Linear form	Eq. no	Plot	Parameters and constants
Pseudo-first-order kinetic	ln (q_e_ − q_t_) = ln q_e_ − k_1_t	(5)	ln(q_e_ − q_t_) vs. t	k_1_ is the pseudo-first-order adsorption rate constant; q_e_ is the amount of antibiotic adsorbed at saturation per gram of adsorbent (mg g^−1^), q_t_ is the amount of antibiotic adsorbed at time t per gram of adsorbent (mg g^−1^)
Pseudo second-order kinetic	$\frac{\mathrm{t}}{{\mathrm{q}}_{\mathrm{t}}}=\left[\frac{1}{{\mathrm{k}}_{2}{\mathrm{qe}}^{2}}\right]+\frac{1}{{\mathrm{q}}_{\mathrm{e}}}\mathrm{ t}$	(6)	t/q_t_ vs. t	k_2_ is adsorption rate constant of the pseudo-second-order
Intraparticle diffusion kinetic	q_t_ = k_i_ t^1/2^ + C	(7)	q_t_ vs. t^1/2^	k_i_ (mg g^-1^ min^−1/2^) is the intraparticle diffusion rate constant, which is the slope of the straight line of *q*_*t*_ versus *t*^1/2^; C is the value of intercept, which is a constant reflecting the significance of the boundary layer or mass transfer effect
Langmuir isotherm	$\frac{{\mathrm{q}}_{\mathrm{e}}}{{\mathrm{C}}_{\mathrm{e}}}=\frac{1}{{\mathrm{K}}_{\mathrm{L}}{\mathrm{q}}_{\mathrm{m}}}+\frac{{\mathrm{C}}_{\mathrm{e}}}{{\mathrm{q}}_{\mathrm{m}}}$	(8)	(C_e_/q_e_) vs. C_e_	q_e_ is the solid-phase concentration in equilibrium with the liquid-phase; concentration C_e_ is expressed in mole L^−1^; q_m_ is the maximum monolayer adsorption capacity (mg g^−1^); and K_L_ is an equilibrium constant (L mol^−1^)
Freundlich isotherm	${\mathrm{lnq}}_{\mathrm{e}}=\mathrm{ ln}{\mathrm{K}}_{\mathrm{f}}+\frac{1}{\mathrm{n}}\mathrm{ ln}{\mathrm{C}}_{\mathrm{e}}$	(9)	ln q_e_ vs. ln C_e_	plotting ln q_e_ versus ln C_e_ gives a straight line with slope of 1/n, where n is a constant related to adsorption intensity and its magnitude shows an indication of the favorability of adsorption; the intercept is ln K_f_ where K_f_ is constant (function of energy of adsorption and temperature)

### Reusability test

To investigate the reusability of PVA/IC/SZrO_2_ composite bead, 10 mg of beads was mixed with 50 mL of 20 mg L^−1^ antibiotic. The mixture was shaken for 15 min. after adsorption, the beads was separated. Then 0.01 M NaOH was added and the solution was shaken again for 15 min. Finally, the beads was repeatedly washed by 70% (v/v) ethanol aqueous solution, until antibiotic was no longer detected in the solution. The cyclic adsorption–desorption processes were conducted up to 10th cycle successfully to study the reusability of the nanocomposite.

## Results and discussion

### Morphological, physical and thermal characteristics of SZrO_2_ and nanocomposite beads

**Figure 1:**
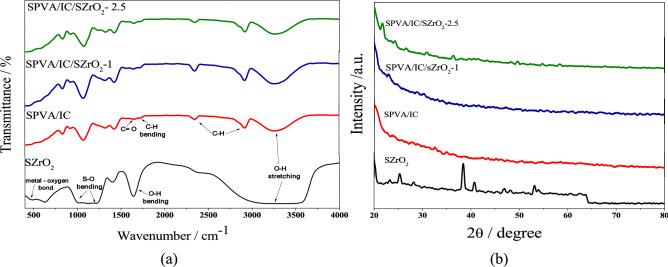
(**a**) FTIR spectra of SZrO_2_ and SPVA/IC/SO_4_ZrO_2_ composite beads, (**b**) XRD patterns of SZrO_2_ and SPVA/IC/ SZrO_2_ composite beads.

Figure 1a illustrate FTIR spectra of SZrO_2_ and PVA/IC/SZrO_2_ composite beads. SZrO_2_ spectrum shows a board peak at 3250 cm^−1^ and sharp peak at 1630 cm^−1^, that may be referred to the adsorbed moisture and hydrogen bonds formed, however, the peak around 500 cm^−1^ return to Zr–O bond, while, peaks at 1217, 1128 and 1016 cm^−1^ are characteristic for S–O. On the other hand, the FTIR spectra for PVA/IC/ SZrO_2_ nanocomposite beads, the bands around 3250 cm^−1^ are attributable to the O–H bonds from water molecules that become more adsorbed as the concentration of sulfated zirconia increases due to its hydrophilic properties, and the bands at 1600 cm^−1^ are indicative of –OH groups of PVA and IC. The sulphate groups of iota carrageenan have a distinctive peak at 830 cm^−1^. The C–H bonds in the polymers structure can be attributed to bands at 2840 and 2300 cm^−1^^25^, while the weak bands at 1700 and 1750 cm^−1^ correspond to C=O bonds and C–H bending, respectively in the aromatic structure of sulfophithalic acid (SPA), which demonstrate that the crosslinking process has been completed. Sulfate groups of SZrO_2_ are responsible for the bands at 950 and 1100 cm^−1^.

In Fig. 1b it can be observed the amorphous structure for the prepared composite beads increased with increasing SZrO_2_ concentration, while the sulfated zirconia powder curve shows characteristic peaks intensity of SZrO_2_ at a 2θ angle 54, 28, 38^26,27^.

**Figure 2:**
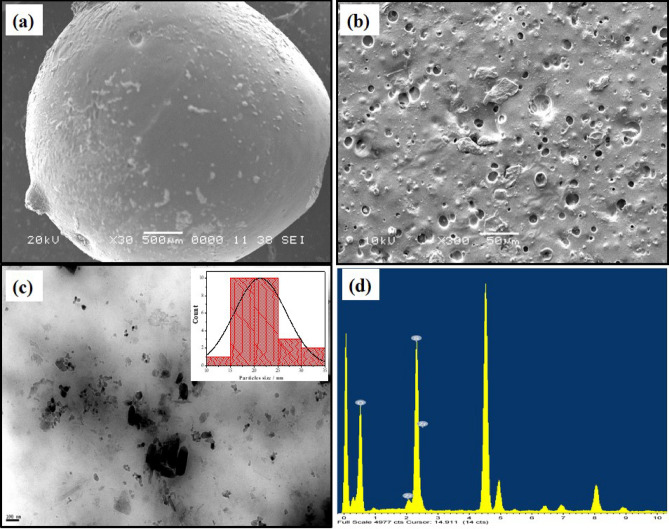
SEM images for (**a**) SPVA/IC/ SZrO_2_-2.5 beads surface, (**b**) SPVA/IC/ SZrO_2_-2.5 inside, (**c**) TEM image for SZrO_2_ nanoparticles with the inset frequency distribution plot of SZrO_2_ nanoparticles size from TEM image, (**d**) EDX analysis for SZrO_2_.

Figure 2a shows a semi-spherical shape for SPVA/IC/SO_4_ZrO_2_ composite beads and porous future for inside surface Fig. 2b. While the TEM image of sulfated zirconia in Fig. 2c demonstrated that, the material developed in nanoscale particles. EDX spectra as shown in Fig. 2d confirmed the presence of sulphur groups on the surface of SZrO_2_, proving the synthesize of sulfated zirconia.

**Figure 3:**
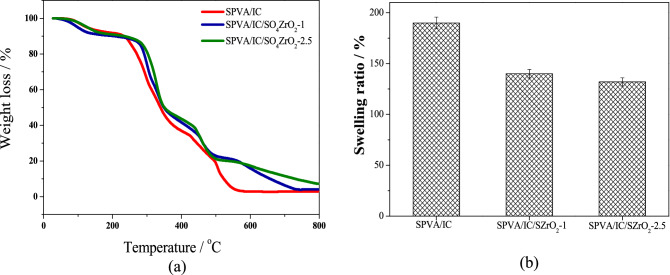
(**a**) TGA curves and (**b**) time dependent swelling response of SPVA/IC and SPVA/IC/SZrO_2_ nanocomposite beads.

As shown in Fig. 3, the TGA curves of beads without and with SZrO_2_shows about 8% loss in weight at ~ 150 ºC and that returns to the moisture evaporation^21^. The second loss stage of composite beads occurred in the range of 150—270 ℃ and is related to the degradation of functional groups, while the third loss stage is characterized by a remarkable decomposition from 270 to 360 ℃ and is related to the decomposition of polymeric chains, which began at 230 ℃ for the undoped beads and at 270 ℃ with a lower weight percentage for the doped beads. This behaviour demonstrates that the addition of SZrO_2_ improves the composite's temperature stability by boosting covalent, ionic, and hydrogen bonding in the nanocomposite.

As shown in Fig. 3b, response of swelling behavior of SPVA/IC, SPVA/IC/SZrO_2_-1 and SPVA/IC/SZrO_2_-2.5 in water with respect to time was studied at room temperature. After a set amount of time had passed, the degree of swelling was assessed, and the immersion experiment lasted 24 h. SPVA/IC beads showed maximum degree of among other beads with SZrO_2_ and that can be explained by adding SZrO_2_ to the polymeric mixture of SPVA and IC may result in an increase in the amount of hydrogen bonds formed between polymer chains and SZrO_2_, resulting in increased chemical stability in polymer chains.

### Adsorption process evaluation

#### Effect of solution pH and adsorption surface chemistry

**Figure 4:**
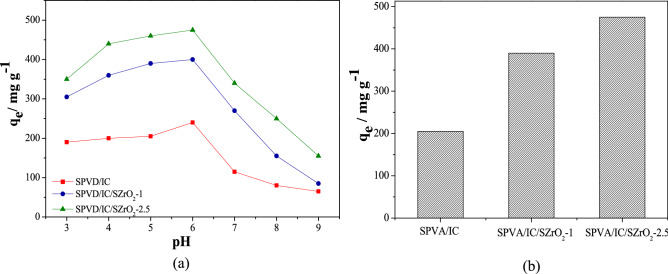
(**a**) Effect of solution's pH (0.01 g adsorbent beads, 50 mL of 100 mg L^−1^ *Cefotaxime* solution at 3 h adsorption time, and 25 °C temperature), (**b**) effect of SZrO_2_ concentration on *Cefotaxime* adsorption process.

It is crucial to investigate the beads' main surface charge at a certain pH. The calculation of the point of zero charge (pH_PZC_) is used for that purpose in addition, it can be used to comprehending the electrostatic interactions of the beads with *Cefotaxime* molecules. The point of zero charge of SPVA/IC nanocomposite beads was found around pH 6.5 while for SPVA/IC/SZrO_2_-1 and SPVA/IC/SZrO_2_-2.5 nanocomposite beads was found around pHs 6.1 and 5.3 respectively as shown in Fig. S3 in supplementary information. However, below these values of PZC the surface become positively charged on the other hand, above these values the surface acquired negative charge for the removal of positive molecules. The SZrO_2_ affects the PZC of SPVA/IC polymeric matrix and due to the greater percent of oxygenated groups content in SZrO_2_ lowers the value of PZC to 6.1 and 5.3 for SPVA/IC/SZrO_2_-1 and SPVA/IC/SZrO_2_-2.5 nanocomposite beads respectively. Two processes are involved in the absorption of *Cefotaxime* from aqueous medium by nanocomposite beads. The first is the diffusion of *Cefotaxime* into nanocomposite beads with water molecule penetration, and the second is associated with electrostatic attractive forces. The pH has a significant and observable impact on the absorption process. The influence of the pH of the solution on *Cefotaxime* adsorption process was studied in the range of 3 to 9 to find the optimal pH value, as shown in Fig. 4a. The results indicate the maximum adsorption has occurred at pH6. The significant decrease of the adsorption capacity at pH ranged between7and 9 may be due to the degradation of *Cefotaxime* followed by hydrolysis of the β-lactam ring and the acetoxy ester. In addition, increasing pH elevate the deprotonation of *Cefotaxime*, accordingly that will increase the repulsion between *Cefotaxime* molecules and negatively charged beads above its PZC values. On the other hand, at very low pH values less than 4, the protonation rate of *Cefotaxime* amine groups will increased, which increases its solubility and decreases its adsorption rate, besides the surfaces of SPVA/IC/SZrO_2_ would be surrounded by many hydronium ions that compete with *Cefotaxime* for active sites. While, the high adsorption capacity at solution pH ranged between 4 and 6 can be referred to maximum stability of *Cefotaxime* at pH ranged between 4 and 6 (pKa for *Cefotaxime* is 3.1)^28^ and that increase the possibility to form hydrogen bond and electrostatic attraction between nanocomposite beads and *Cefotaxime* molecules as shown in Fig. S4 in supplementary information. Thus, pH6 was selected for further adsorption studies of *Cefotaxime*.

A batch adsorption process was used to investigate the effect of adding SZrO_2_ with different concentrations on *Cefotaxime* adsorption process onto polymeric beads. As shown in Fig. 4b it can be noted that the adsorption capacity of the beads was increased with increasing SZrO_2_ content and that may be referred to forming hydrogen bond and electrostatic attraction force between oxygenated groups of the composite and amino groups in *Cefotaxime*.

#### Effect of contact time

**Figure 5:**
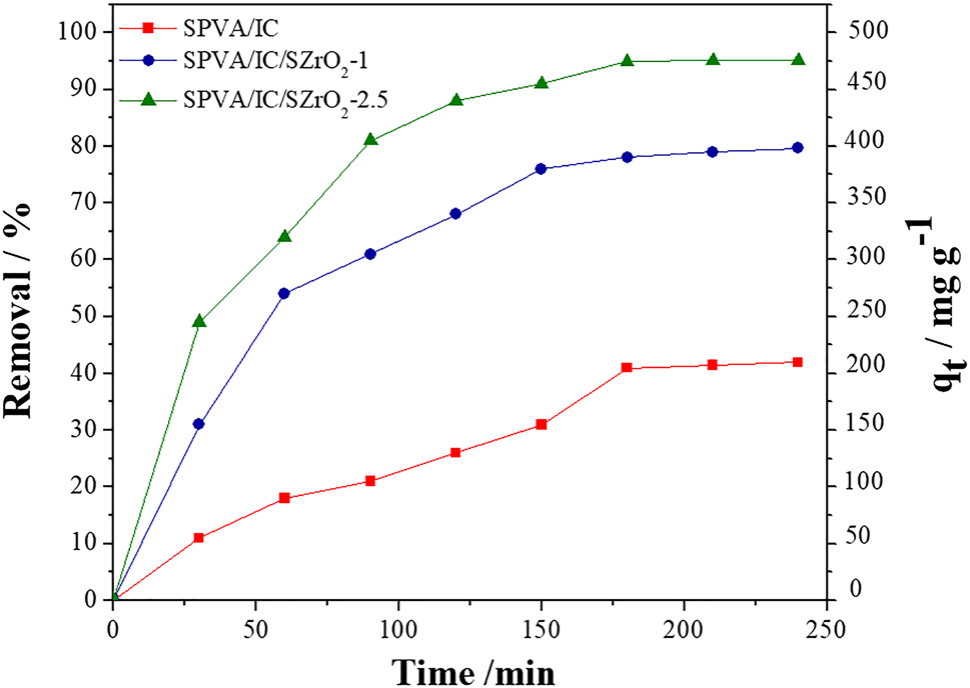
Adsorption kinetics of *Cefotaxime* onto composite beads at C_0_; 100 mg L^−1^, dose of adsorbent beads 0.2 mg mL^−1^, at 25 °C and pH  6.

As shown in Fig. 5**,** for all beads samples, the adsorption capacity grew significantly at first, then gradually increased and eventually became constant over time. This could be due to the fact that there are free surface sites available for adsorption during the initial adsorption stage, but due to repulsive forces between *Cefotaxime* molecules adsorbed on beads and those in the solution, the remaining unoccupied surface sites are difficult to utilize as time passes. However, the adsorption process reached equilibrium after approximately 3 h. Furthermore, it can be also noticed the removal efficiency of *Cefotaxime onto* SPVA/IC/SZrO_2_-2.5 is higher than SPVA/IC and SPVA/IC/SZrO_2_-1 and that may referred to SPVA/IC/SZrO_2_-2.5 contain more active side than the other types of beads.

The RSM is a model based on statistics and it is often used to clarify the interaction between reaction parameters for optimization since it is faster, practical and all of the components' effects and potential interactions may be evaluated. The adsorption of *Cefotaxime* onto SPVA/IC/SZrO_2_-2.5 nanocomposite beads was evaluated according to matrix design illustrated in Table S2 in supplementary information using independent variables including (A) time in hours, (B) *Cefotaxime* concentration in mg L^−1^ and (C) beads dosage in mg on dependent response, which is removal efficiency (%). To explain *Cefotaxime* elimination using SPVA/IC/SZrO_2_-2.5 beads, the following regression equation for the link between response and variables (A, B and C) was utilized:

$$ Removal \, \left( \% \right) \, = 95 \, + 6.21A \, - 11.25B \, + 17.46C \, + 5AB - 2.08AC + 10BC - 9.04A^{2} + 0.5375B^{2} - 13.04C^{2} $$

**Figure 6:**
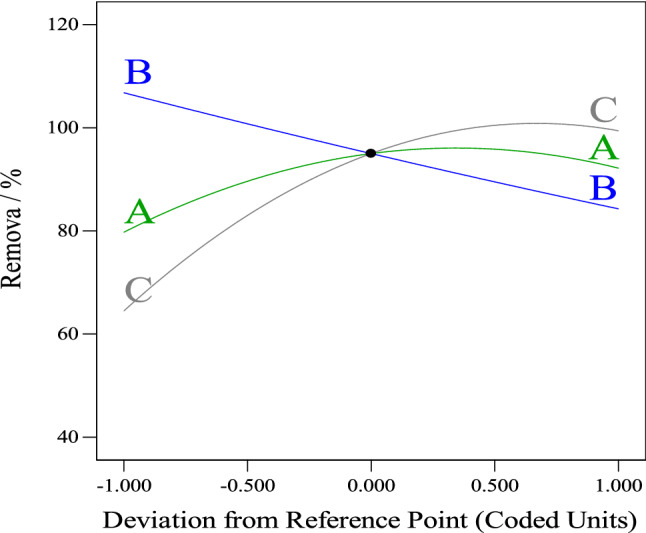
The perturbation plot.

Moreover, according to the perturbation plot for *Cefotaxime* removal efficiency with SPVA/IC/SZrO_2_-2.5 nanocomposite beads as shown in Fig. 6, it was observed that as *Cefotaxime* concentrations increases the removal efficiency decreases, while factors time and beads dose have significant effect on removal efficiency whereas they increase, the removal efficiency increased and then decreased.

ANOVA analysis of variance is well-known for determining the statistical significance of the quadratic response surface model. As shown in Table S3, the quadratic model is very suitable for high coefficient of determination R^2^ (0.9566). The p value of the quadratic model was less than 0.05, therefore, this quadratic model was found to be significant in addition this model is confirmed due to the F-value is found to be 17.16.

**Figure 7:**
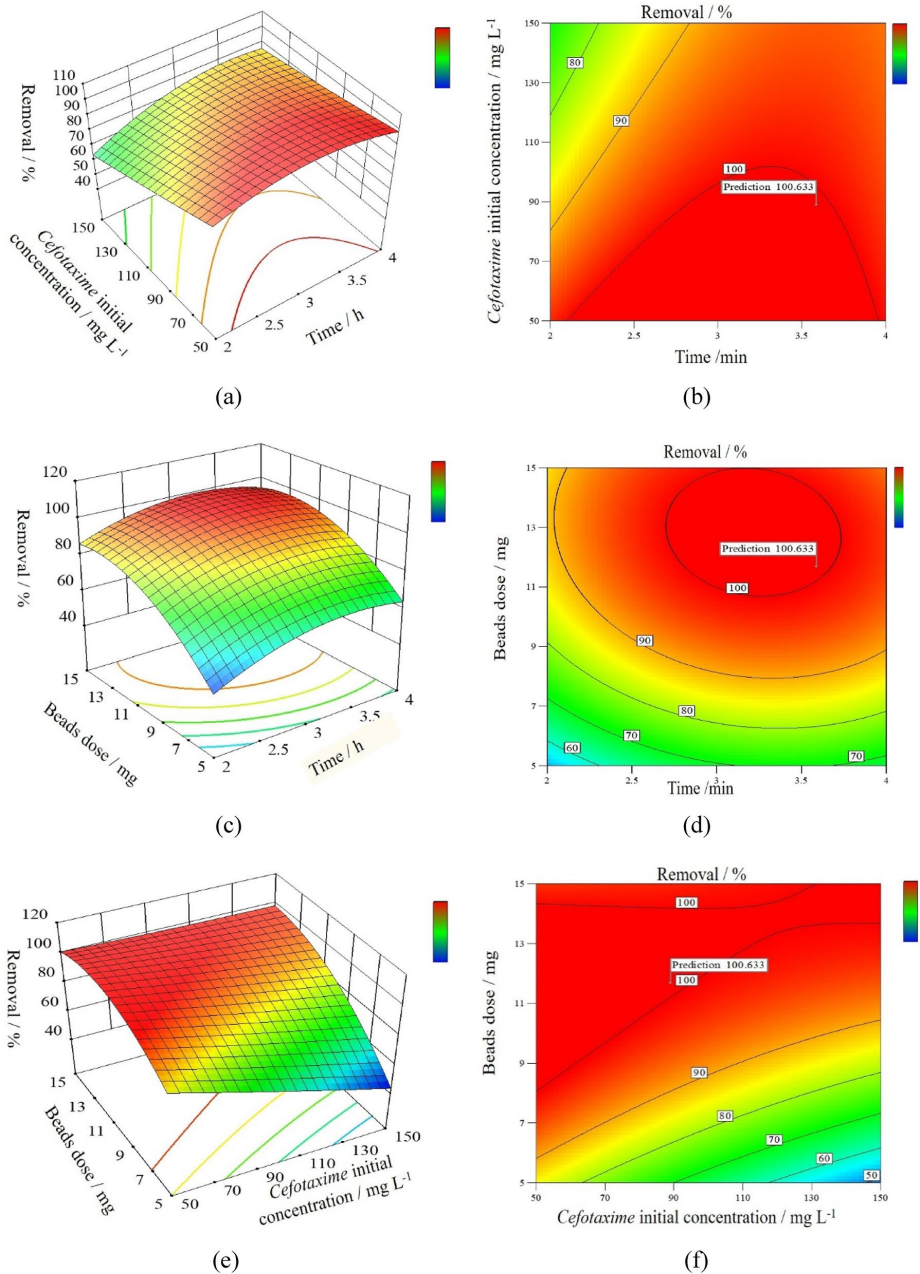
Response surface plots for removal efficiency (%) of *Cefotaxime* onto SPVA/IC/SZrO_2_-2.5 nanocomposite beads (**a,c,e**) 3D surface plots and (**b,d,f**) 2D surface plots.

According to RSM analysis results as shown in Fig. 7, the following are the optimum conditions: initial *Cefotaxime* concentration is 88.97 mg L^−1^ at time: 3.58 h and adsorbent dosage: 11.68 mg to achieve full removal. The efficiency of *Cefotaxime* elimination was discovered to be impacted by all variable parameters.

#### Kinetics and isotherms of adsorption process

Three kinetic models were used to study the adsorption of *Cefotaxime* onto SPVA/IC/SZrO_2_-2.5 nanocomposite beads: pseudo-first-order, pseudo-second-order, and intraparticle diffusion models. For the three kinetic models at varying concentrations, the calculated sorption capacities, reaction rate constants, and R^2^ values of the linear correlation coefficients were shown in Table 2. The sorption capacities estimated using the pseudo-first-order equation were found to be significantly different from the experimental results. While, for the pseudo-second-order kinetic model, the resulting R^2^ values are generally greater than those obtained from pseudo-first-order kinetics, implying that the sorption process followed pseudo-second-order kinetics and that chemisorption was the rate-controlling phase. This appears to include valence forces as a result of chemical reactions or electron exchange between oxygenated groups on *Cefotaxime* molecules and hydroxyl groups on nanocomposite beads and that were responsible for the hydrogen bonds. Despite the fact that the pseudo-second-order-kinetics model had the best fit order, the findings produced from this model were insufficient to evaluate *Cefotaxime* molecules' diffusion mechanism onto SPVA/IC/SZrO_2_-2.5 nanocomposite beads. As shown in Fig. S5 in supplementary information, the adsorption process linear fitting findings were consistent with the intraparticle diffusion model, whereas the diffusion mechanism was split into three stages that could be explained by the following. At the beginning *Cefotaxime* molecules transport through the solution to the bead surface and form film diffusion at the boundary layer, or charged molecules diffuse from a bulk solution to the beads' exterior surface after that the *Cefotaxime* charged molecules transferred into the pores and intraparticular active sites of beads. Finally, the *Cefotaxime* molecules were chemically bound via small pores in the beads, followed by the final equilibrium adsorption.

**Table 2:** Kinetic models’ parameters and determination coefficients for *Cefotaxime* adsorption onto SPVA/IC/SZrO_2_-2.5 nanocomposite beads.

	*Cefotaxime* initial concentrations (mg L^−1^) adsorbed onto SPVA/IC/SZrO_2_-2.5 nanocomposite beads		
	50	100	150
q_e,exp_ (mg g^−1^)	246	475	659
**Pseudo-1st-order**			
q_e'cal_ (mg g^−1^)	249.98	476.6	664.1
k_1_(min^−1^)	0.031	0.031	0.035
R^2^	0.76	0.94	0.85
**Pseudo-2nd-order**			
q_e,cal_ (mg g^−1^)	245.8	476.1	658.7
k_2_(min^−1^)	0.0038	0.0019	0.0013
R^2^	0.989	0.981	0.972
**Intraparticle diffusion model**			
k_i_			
k_i,1_	15.16	31.13	64.48
k_i,2_	12.88	18.24	17.25
k_i,3_	1.89	0.48	2.4
C	45.69	56.84	64.48
R^2^	0.92	0.87	0.94

Langmuir and Freundlich models as shown in Fig. S6 were explored at different beginning concentrations and three different temperatures (25, 35 and 45 ºC), as illustrated in Table 3. The high adsorption capacity showed a strong electrostatic contact between *Cefotaxime* molecules and the bending sites of the beads, and the R^2^ values of the Langmuir model were extremely near to one. Furthermore, at low temperatures, R_L_ values were higher than at high temperatures, and its values were found to be between zero and one, indicating that the adsorption process is chemical rather than physical^22^. However, the results showed that the adsorption process onto beads was best fitted by Freundlich isotherm model, with values of "n" ranging from 2 to 10, which indicate good adsorption and implying higher efficiency for *Cefotaxime* adsorption by nanocomposite beads as the values get increase.

**Table 3:** Adsorption isotherm parameters for *Cefotaxime* adsorption onto SPVA/IC/SZrO_2_-2.5 nanocomposite beads at adsorbent dosage 0.2 mg mL^−1^.

Temperature ºC	Ciprofloxacin adsorption onto FMFNs		
	20 ºC	30 ºC	40 ºC
**Langmuir isotherm**			
q_m_ (mg g^−1^)	735	752	752
k_L_(L mg^−1^)	0.49	0.57	0.98
R^2^	0.992	0.997	0.999
R_L_	0.1	0. 008	0.005
**Freundlich isotherm**			
K_F_ (mg g^−1^)	270	299	330
1/n_F_	0.32	0.34	0.31
R^2^	0.99	0.96	0.85

#### Desorption–adsorption test

**Figure 8:**
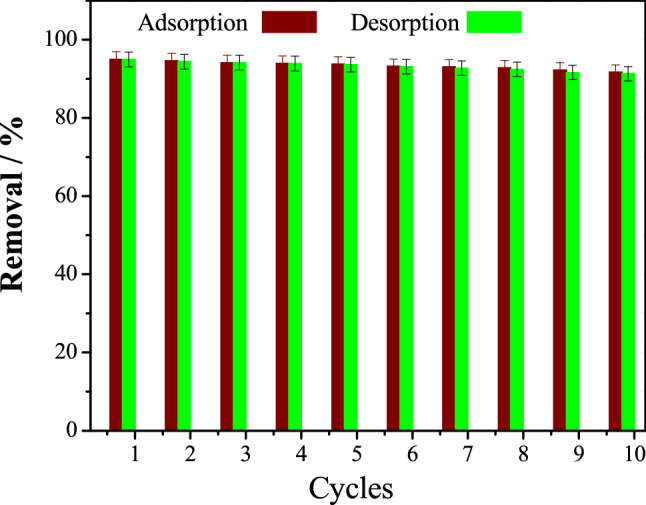
Adsorption–desorption cycles.

After 10 cycles, the adsorbent's ability to regenerate was tested, and it was observed that the removal efficiency was still high, and that the fluctuations in the removal % after each desorption cycle were quite minor, as shown in Fig. 8, indicating that SPVA/IC/SZrO_2_-2.5 nanocomposite beads had potential in wastewater application.

The efficiency of prepared SPVA/IC/SZrO_2_-2.5 nanocompoaite beads for drug removal was compared with other adsorbent materials as illustrated in Table 4.

**Table 4:** A review of the performance of some nanocomposite adsorbent materials used to remove drug from water.

Adsorbent nanomaterials	Adsorbate	Optimum adsorption condition	Adsorbate initial concentration (mg L^−1^)	Maximum adsorption capacity (mg g^−1^)	Reusability cycles/removal% after last cycle	References
Artich-Bch-NaOH	Metformin hydrochloride (MFH)	Acidic medium, 45 min	10	~ 17	5 cycles, 78.5%	^29^
NBent-NTiO_2_-Chit nanocomposite	Levofloxacin (LEVO) Ceftriaxone (CFT)	pH4, 10 min pH5, 10 min	5	~ 43 ~ 36	3 cycles, 95% 3 cycles, 92.8%	^30^
NFe_3_O_4_@Zn(GA)/Starch-Hydrogel	Fluvastatin (FLV)	pH2, 30 min	40	~ 782	5 cycles, 700 mg g^−1^	^31^
V_2_O_5_@Ch/Cu-TMA nanobiosorbent	levofloxacin (LEVO)	pH3, 30 min	10	~ 9	4 cycles, 84.35%	^32^
Fe_3_O_4_–MoO_3_-AC	Ciprofloxacin (CPF)	pH7, 30 min	10	~ 19	5 cycles, 90.5%	^33^
SPVA/IC/SZrO_2_-2.5 nanocompoaite	Cefotaxime	pH6, 3 h	100	~ 475	10 cycles, 91%	This work

## Conclusions

Novel and low expensive nanocomposite beads was produced using a simple blending, eco-environmentally approach and available polymers as PVA and IC. It was appeared that the incorporation of different weight ratio of SO_4_ZrO_2_ as doping agent into the polymeric PVA/IC blend improves the nanocomposite beads adsorption capacity towards *Cefotaxime* antibiotic. It was discovered that when the ultimate concentration of 100 mg L^−1^ *Cefotaxime* solution was compared at varied contact times and pH 6 conditions, the outcomes were better in the case of SPVA/IC/SZrO_2_-2.5 nanocomposite beads. The increasing of SO_4_ZrO_2_ content in the PVA/IC matrix increases porosity and active functional groups such as oxygen containing groups, so that the adsorption efficiency improves. At various experimental conditions, the time-dependent adsorption capacity was assessed. According to the experimental optimization, it was observed that as *Cefotaxime* concentrations increases the removal efficiency decreases, while factors time and beads dose have significant effect on removal efficiency whereas they increase, the removal efficiency increased and then decreased. The adsorption kinetic of *Cefotaxime* was better fitted to pseudo-second-order model, and the adsorption isotherm was better fitted to the Langmuir and Freundlich models. The ability of SPVA/IC/SZrO_2_-2.5 nanocomposite beads to regenerate ten times further demonstrated its suitability for long-term use. The current study reveals that SPVA/IC/SZrO_2_-2.5 nanocomposite beads have a lot of potential in water treatment for removing antibiotic pollutants because of their fast separation, ease of operation, high capture capacity, and good cycle performance.

## Supplementary Information

Supplementary Information.

## Data Availability

All data generated or analyzed during this study are included in this published article and its supplementary information files.
