# Supplementary material for: Development of hybrid green nanocomposite polymeric beads doped with nano sulfated zirconia for effective removal of Cefotaxime antibiotic from aqueous solution
Source: Sci Rep. 2022 Jul 26;12:12701. doi: 10.1038/s41598-022-16473-z (PMC9325701; doi:10.1038/s41598-022-16473-z)
Supplement: Supplementary file 1 — Supplementary Information. [file 41598_2022_16473_MOESM1_ESM.pdf]

Supplementary information

Development of Hybrid Green Nanocomposite Polymeric Beads Doped with Nano Sulfated Zirconia for Effective Removal of Cefotaxime antibiotic from Aqueous Solution

Marwa H. Gouda[1] Noha A. Elessawy[2]*, Arafat Toghan[3, 4]

[1] Polymer Materials Research Department, Advanced Technology and New Materials Research Institute (ATNMRI), City of Scientific Research and Technological Applications City (SRTA-City), 21934 Alexandria, Egypt.

[2] Computer Based Engineering Applications Department,Informatics Research Institute IRI, City of Scientific Research and Technological Applications City (SRTA-City), 21934 Alexandria, Egypt.

[3]Chemistry Department, Faculty of Science, South Valley University, Qena 83523, Egypt.

[4]Chemistry Department, College of Science, Imam Mohammad Ibn Saud Islamic University (IMSIU), Riyadh 11623, Saudi Arabia.

Corresponding author E-mail addresses: (Noha A. Elessawy): nony_essawy@yahoo.com

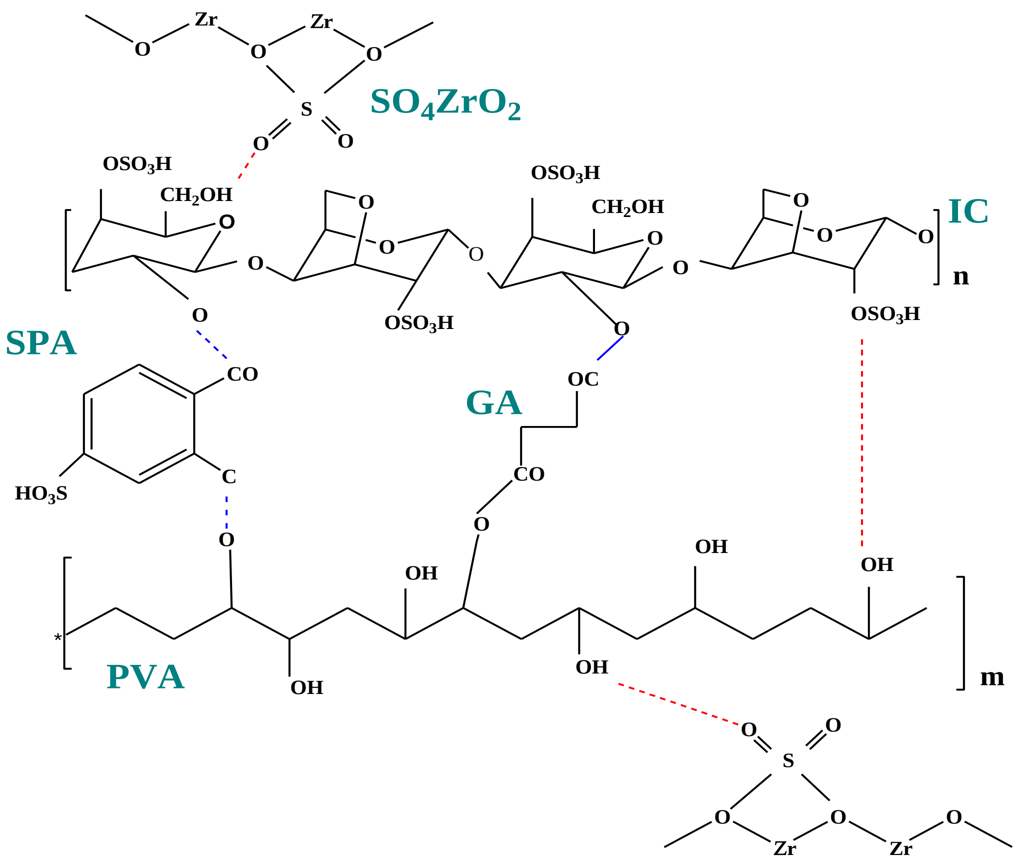

Figure S1: Possible structure of the SPVA/ IC/ $SZrO_2$ nanocomposite beads

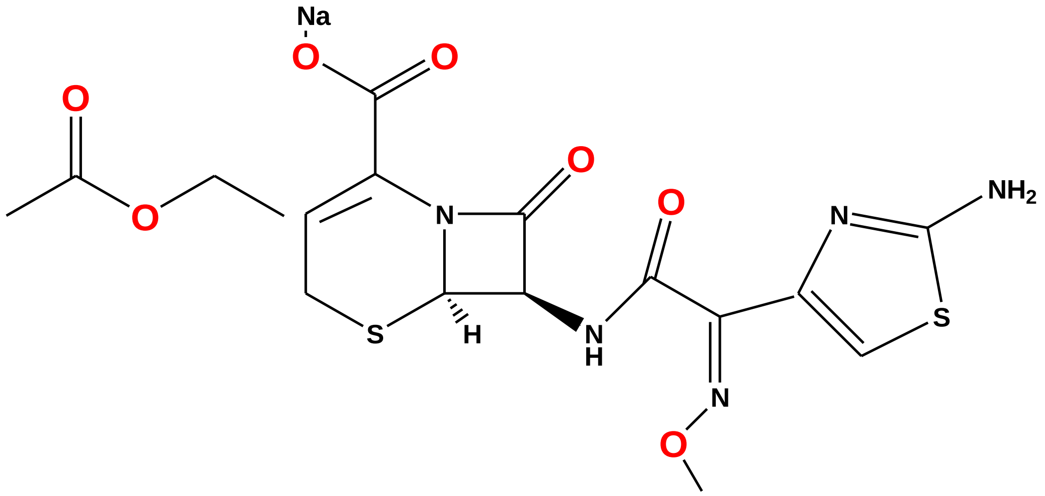

Figure S2: chemical structure of *Cefotaxime* antibiotic molecules.

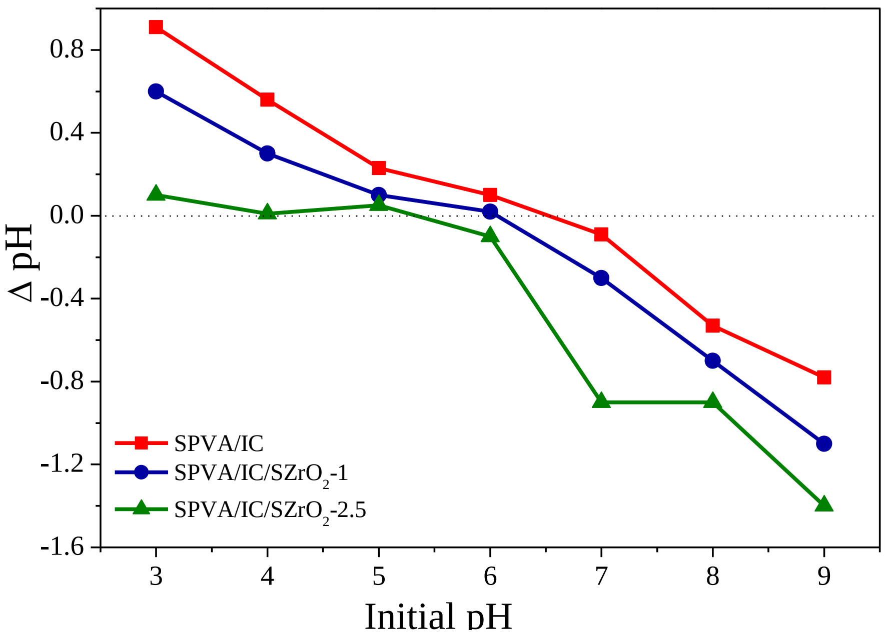

Figure S3: point of zero charge curve of PVA/IC/PANI/GO nanofiber composite membrane.

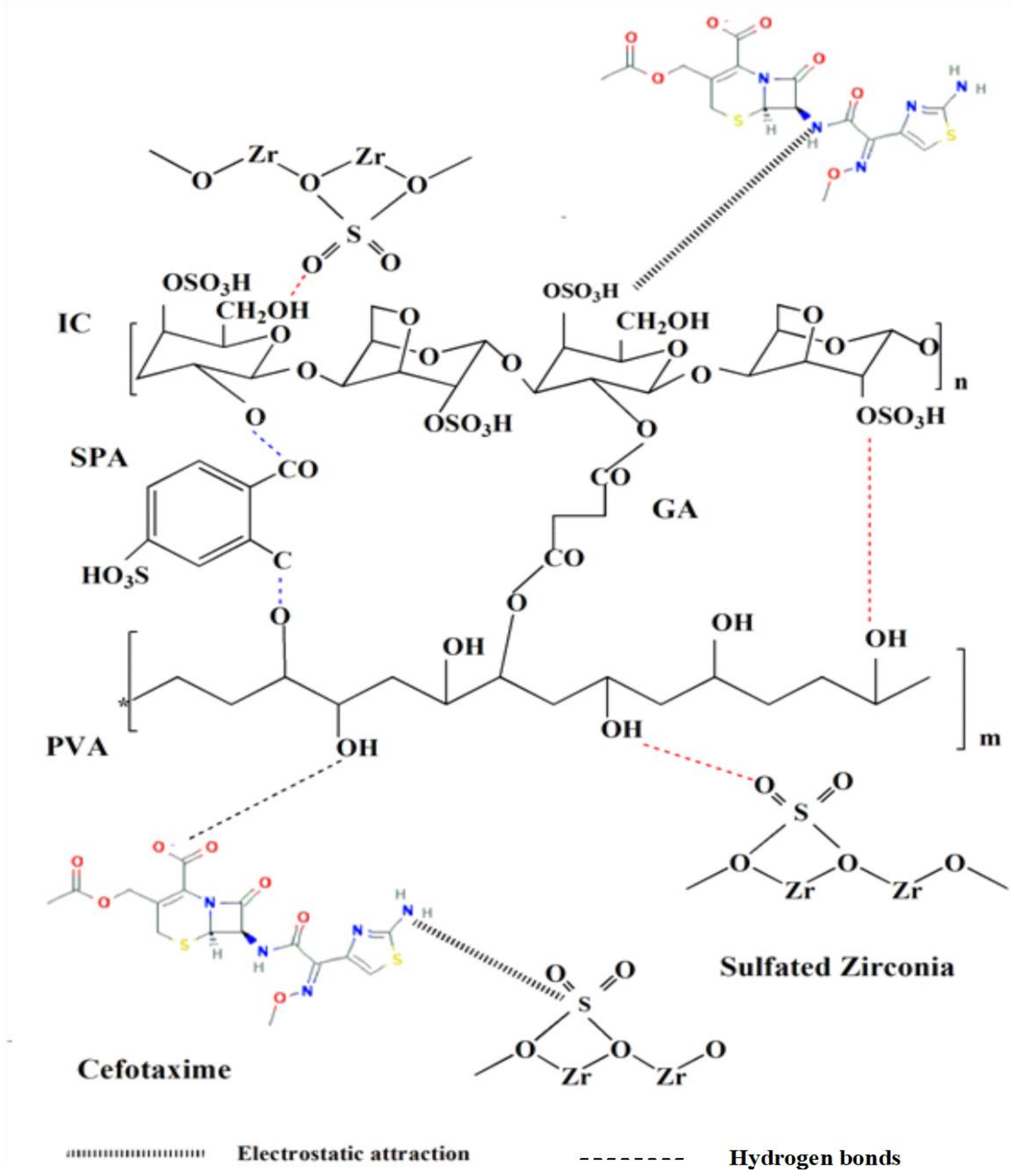

Figure S4: The possible interaction of absorption between SPVA/IC/$SZrO_2$ beads and *Cefotaxime* molecules.

(a)

(b)

(c)

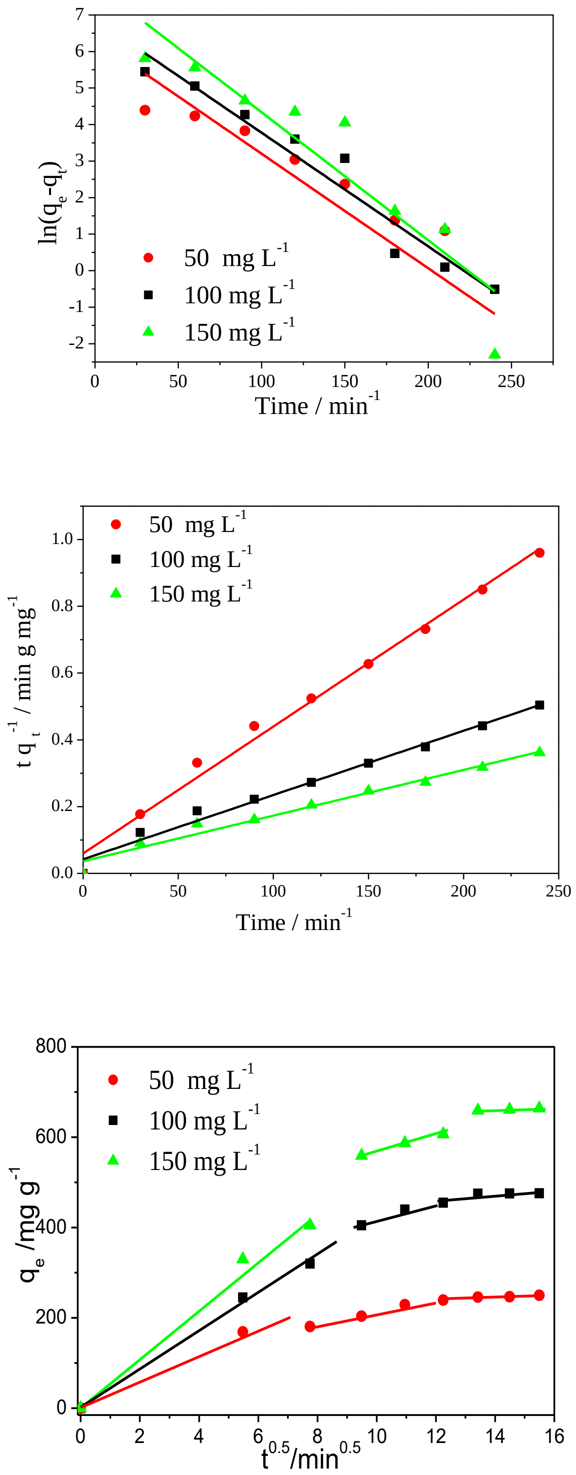

Figure S5: (a) pseudo-first-order, (b) pseudo-second-order and (c) intraparticle diffusion kinetic models fit for *Cefotaxime* adsorption onto SPVA/IC/SZrO_2-2.5 nanocomposite beads

(a)

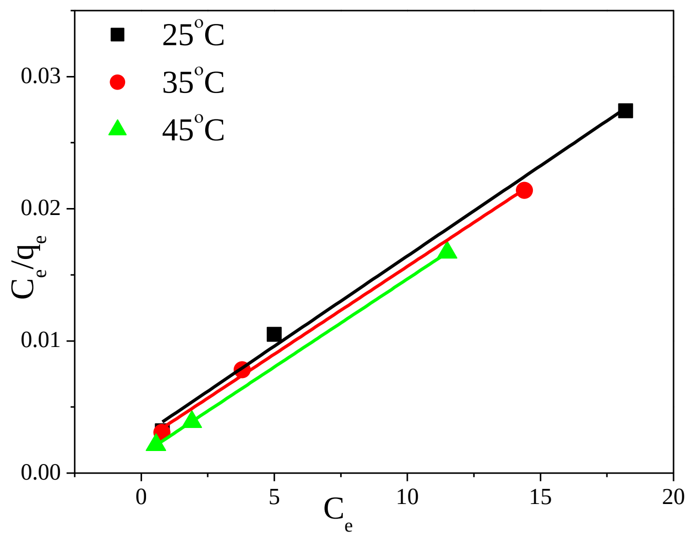

(b)

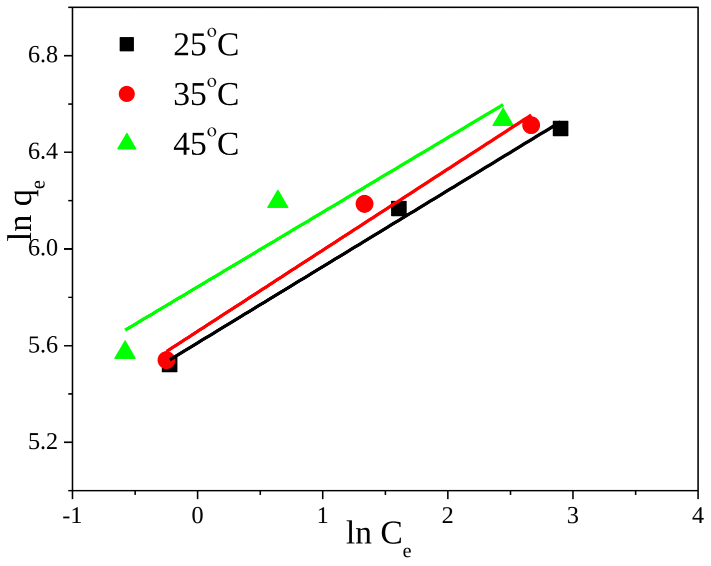

Figure S6: (a) Langmuir and (b) Freundlich isotherm models fit for Cefotaxime adsorption onto SPVA/IC/SZrO_2-2.5 nanocomposite beads

Table S1: Level of various independent variables at coded values of response surface methodology experimental design

Symbol	Independent variables	Coded levels		
		-1	0	1
A	Time /h	2	3	4
B	initial antibiotic concentration / mg L^{-1}	50	100	150
C	adsorbent dose / mg	5	10	15

Table S2: The Box-Behnken design matrix and results for the three variables that influenced the adsorption of *Cefotaxime* onto SPVA/IC/SZrO_2-2.5 nanocomposite beads

**Trial**	**Time (A; hour)**	***Cefotaxime* initial concentration (B;mg L^{-1})**	**Adsorbent dose (C;mg)**	**Removal (%)**	
				**Measured**	**Predicted**
**1**	3	100	10	95	95
**2**	3	150	5	45	43.79
**3**	3	100	10	95	95
**4**	3	100	10	95	95
**5**	4	100	5	59	63.75
**6**	4	100	15	98.7	94.23
**7**	3	50	15	100	101.2
**8**	2	50	10	93	96.54
**9**	2	100	15	91	91
**10**	4	50	10	96	96
**11**	2	100	5	43	47.18
**12**	3	100	10	95	95
**13**	3	100	10	95	95
**14**	2	150	10	67	64.04
**15**	3	150	15	91	98.7
**16**	4	150	10	90	86.46
**17**	3	50	5	94	86.29

Table S3: ANOVA analysis for response function of *Cefotaxime* removal efficiency with $SPVA/IC/SZrO_2$-2.5 nanocomposite beads

Source	Sum of squares	df	Mean square	F-value	p-value
Model	5394.91	9	599.43	17.16	0.0006
A-Time	308.76	1	308.76	8.84	0.0207
B- *Cefotaxime* initial concentration	1012.50	1	1012.50	28.99	0.0010
C- Adsorbent dose	2439.51	1	2439.51	69.85	< 0.0001
AB	100.00	1	100.00	2.86	0.1345
AC	17.22	1	17.22	0.4931	0.5052
BC	400.00	1	400.00	11.45	0.0117
A^2	343.90	1	343.90	9.85	0.0164
B^2	1.22	1	1.22	0.0348	0.8572
C^2	715.69	1	715.69	20.49	0.0027
Residual	244.47	7	34.92		
Lack of Fit	244.47	3	81.49		
Pure Error	0.0000	4	0.0000		
Cor Total	5639.38	16			

**Std. Dev.**	5.91	**R^2**	0.9566
**Mean**	84.86	**Adjusted R^2**	0.9009
**C.V. %**	6.96	**Predicted R^2**	0.3064
		**Adeq Precision**	12.6695
